# Correction to “Autoimmune Thyroid Disease and Female Fertility: Clinical Evidence on Ovarian Reserve, Infertility Treatment Outcomes, and Pathophysiology”

**DOI:** 10.1002/rmb2.70065

**Published:** 2026-06-10

A. Iwase, Y. Hasegawa, Y. Kitahara, et al., “Autoimmune Thyroid Disease and Female Fertility: Clinical Evidence on Ovarian Reserve, Infertility Treatment Outcomes, and Pathophysiology,” *Reproductive Medicine and Biology* 25, no. 1 (2026): e70052, https://doi.org/10.1002/rmb2.70052.

The author names “Harada Miyuki” and “Kawamura Kazuhiro” were incorrectly presented with the surname and given name in the wrong order. The correct format (First name, Last name) should be “Miyuki Harada” and “Kazuhiro Kawamura”.

We apologize for this error.

